# Risk Assessment of Cnm-Positive *Streptococcus mutans* in Stroke Survivors (RAMESSES): Protocol for a Multicenter Prospective Cohort Study

**DOI:** 10.3389/fneur.2022.816147

**Published:** 2022-05-12

**Authors:** Satoshi Hosoki, Yorito Hattori, Satoshi Saito, Misa Takegami, Shuichi Tonomura, Yumi Yamamoto, Shuhei Ikeda, Naohisa Hosomi, Naoya Oishi, Yoshiaki Morita, Yoshihiro Miyamoto, Ryota Nomura, Kazuhiko Nakano, Masafumi Ihara

**Affiliations:** ^1^Department of Neurology, National Cerebral and Cardiovascular Center, Suita, Japan; ^2^Department of Preventive Medicine and Epidemiology, National Cerebral and Cardiovascular Center, Suita, Japan; ^3^Department of Neurology, Kyoto University Graduate School of Medicine, Kyoto, Japan; ^4^Department of Molecular Innovation in Lipidemiology, National Cerebral and Cardiovascular Center, Suita, Japan; ^5^Department of Neurology, Chikamori Hospital, Kochi, Japan; ^6^Department of Disease Model, Research Institute of Radiation Biology and Medicine, Hiroshima University, Hiroshima, Japan; ^7^Medical Innovation Center, Kyoto University Graduate School of Medicine, Kyoto, Japan; ^8^Department of Radiology, National Cerebral and Cardiovascular Center, Suita, Japan; ^9^Department of Medical and Health Information Management, National Cerebral and Cardiovascular Center, Suita, Japan; ^10^Department of Pediatric Dentistry, Osaka University Graduate School of Dentistry, Suita, Japan

**Keywords:** small vessel disease (SVD), cerebral microbleeds (CMBs), intracerebral hemorrhage (ICH), Cnm, *Streptococcus mutans* (*S. mutans*), dental caries, stroke

## Abstract

**Introduction:**

The role of commensal microbiota in systemic diseases, including brain diseases, has attracted increasing attention. Oral infectious diseases, such as dental caries and periodontitis, are also involved in cerebrovascular diseases and cognitive impairment. Cerebral microbleeds (CMBs) and intracerebral hemorrhage due to small vessel disease (SVD), are presumably associated with a high risk of vascular cognitive impairment and stroke. We previously reported that *Streptococcus mutans* (*S. mutans*, the main pathogen of dental caries), harboring the *cnm* gene that encodes the collagen-binding protein Cnm, is associated with the development of hypertensive intracerebral hemorrhage and aggravation of CMBs. We also proposed a mechanism by which the circulating Cnm-expressing *S. mutans* causes intracerebral hemorrhage or CMBs; it binds to denuded basement membranes mainly composed of collagen IV through damaged tight junctions or it directly invades endothelial cells, resulting in blood-brain barrier injury. In November 2018, we initiated a multicenter, prospective cohort study (RAMESSES: Risk Assessment of Cnm-positive *S. mutans* in Stroke Survivors; UMIN Clinical Trials Registry: UMIN000045559) to explore the longitudinal association between Cnm-positive *S. mutans* and CMBs with comprehensive dental findings, which should determine the effect of Cnm-positive *S. mutans* in the oral cavity on the risk of CMB development and cognitive decline.

**Methods:**

Fifteen domestic institutes will be enlisted to enroll 230 patients who have at least one CMB in the deep brain area and develop a stroke within the past year. The prevalence of Cnm-positive *S. mutans* based on oral specimens and dental hygiene will be examined. The primary outcome is the number of newly developed deep CMBs. The secondary outcomes include the new development of lobar, subtentorial, or any type of CMBs; symptomatic intracerebral hemorrhage or ischemic stroke; changes in cognitive function or frailty; major bleeding; all-cause mortality; and antibody titers against periodontal pathogens. The observation period will be 2 years.

**Discussion:**

The 2-year longitudinal prospective cohort study is expected to establish the role of Cnm-positive *S. mutans* in SVD including CMBs and intracerebral hemorrhage from the perspective of the “brain-oral axis” and provide guidance for novel prophylactic strategies against Cnm-positive *S. mutans*-induced SVD.

## Introduction

Small vessel disease (SVD) refers to a variety of pathological, clinical, and neuroimaging changes that are caused by damage to the perforating cerebral arterioles, capillaries, and venules. Vascular pathologies underlying SVD include lipohyalinosis, fibrinoid necrosis, microatheroma, microaneurysms, and segmental arterial disorganization (1, 2). White matter hyperintensities (WMHs), lacunes, perivascular spaces, and cerebral microbleeds (CMBs) detected by brain magnetic resonance imaging (MRI) are major neuroimaging markers of SVD (3, 4). Among them, CMBs appear as small circular or elliptical lesions with a size of 10 mm or less that have a low signal intensity on gradient-echo T2^*^-weighted images (T2^*^WI) obtained by brain MRI (5–7). In addition, CMBs are an independent risk factor for cognitive impairment (8) and stroke (9), especially intracerebral hemorrhage (ICH) (10). It is well-known that the vascular risk factors, including hypertension, diabetes, dyslipidemia, and smoking, play important roles in developing and exacerbating SVD mediated by oxidative stress and vascular inflammation resulting in endothelial damage and disruption of the blood-brain barrier (BBB) (1). However, the effect of vascular risk factors on WMHs may be limited because WMHs have a substantial “non-vascular” or non-atheromatous etiology (4). This notion may be supported by the fact that the modification of vascular risk factors is not sufficient for the prevention and treatment of SVD.

Numerous studies have found an association between human microbiota and many pathological conditions, including cardiovascular diseases, diabetes, obesity, rheumatoid arthritis, Alzheimer's disease, and multiple sclerosis, which lead to the elucidation of the molecular mechanisms underlying this association (11). Specifically, the relationship between oral infections and cardiovascular diseases has been examined in several observational studies (12) and interventional studies (13). However, it remains to be fully elucidated which oral bacteria among more than 700 species are involved and how these causative agents exert the deleterious effects that induce systemic pathological changes (14). *Streptococcus mutans* (*S. mutans*) is a gram-positive bacterium and a major pathogen responsible for dental caries (15). Cnm, encoded by the *cnm* gene, is a cell-surface 120-kDa collagen-binding protein of *S. mutans* (16). Intravenous administration of Cnm-expressing *S. mutans* (Cnm-positive *S. mutans*) aggravates ICH in stroke-prone spontaneously hypertensive rats and cerebrovascular-endothelial-injured mice (17). We also reported that oral carriage of Cnm-positive *S. mutans* is associated with the prevalence of deep (basal ganglia and thalamus) CMBs and ICH (odds ratio: 4.5 vs. ischemic stroke) (18), and it is linked to an increased deep CMB incidence (incidence rate ratio: 13.9), based on cross-sectional studies (19), and to the severity of deep CMBs according to a longitudinal retrospective study (20). Cnm-positive *S. mutans* is also associated with cognitive impairment, accompanied by increased CMBs (19, 21).

**Figure 1:**
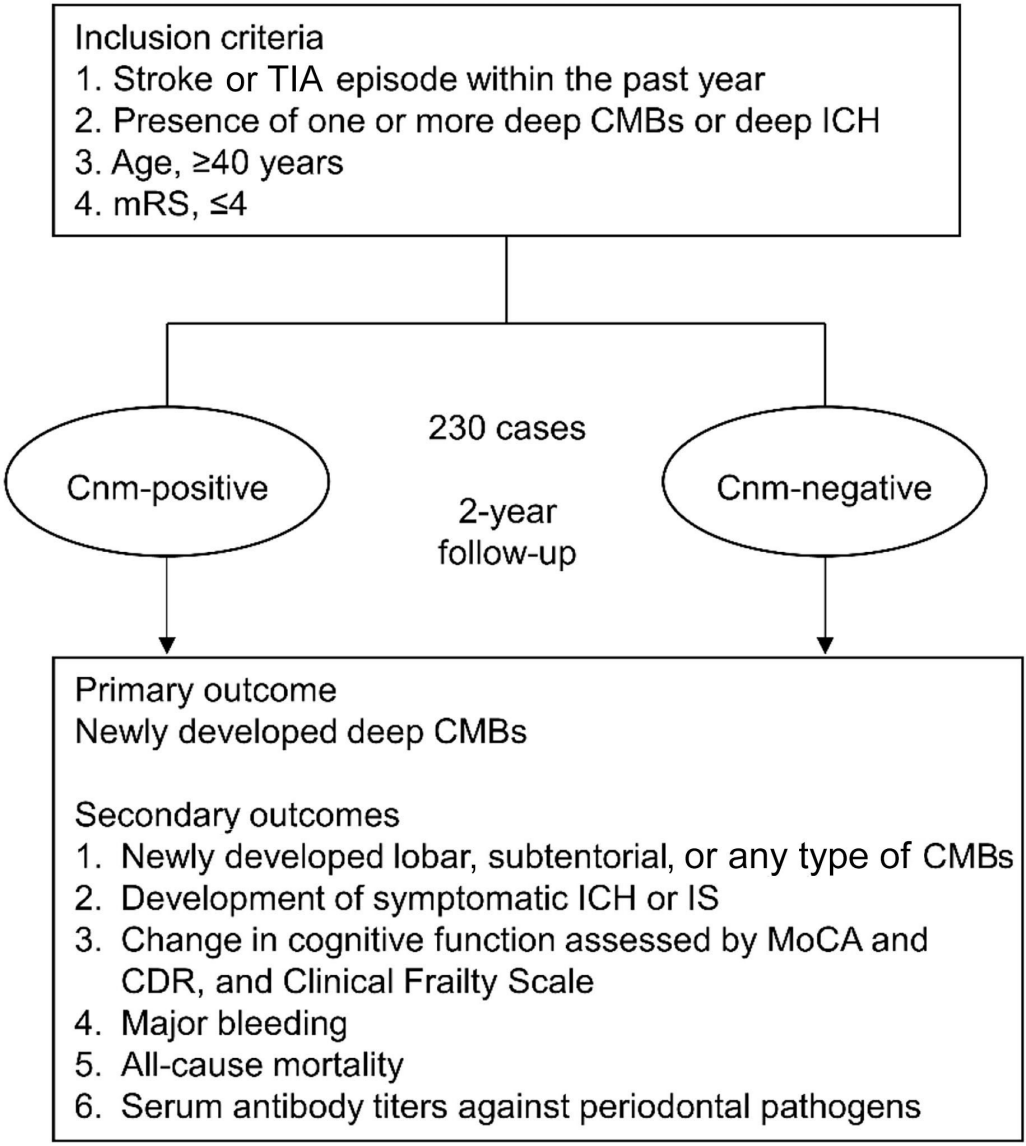
Research protocol of the RAMESSES study. The RAMESSES study is a multicenter, prospective, longitudinal, observational study to investigate the role of Cnm-positive *Streptococcus mutans* in the development of SVD and vascular cognitive impairment. Fifteen Japanese institutes participate in this study using a standardized MRI protocol. TIA, transient ischemic attack; CMBs, cerebral microbleeds; mRS, modified Rankin Scale; ICH, intracerebral hemorrhage; IS, ischemic stroke; MRI, magnetic resonance imaging; MoCA, Montreal Cognitive Assessment; CDR, clinical dementia rating.

Thus, cross-sectional and retrospective studies have identified strong associations of Cnm-positive *S. mutans* with ICH, deep CMBs, and cognitive dysfunction. However, the long-term effects of Cnm-positive *S. mutans* on SVD remain to be elucidated. In addition, the relationship between Cnm-positive *S. mutans* and oral hygiene and periodontal disease remains unknown. Therefore, in November 2018, we initiated a multicenter, prospective cohort study named RAMESSES (Risk Assessment of cnM-positivE S. mutans in StrokE Survivors) to explore the longitudinal association between Cnm-positive *S. mutans* and SVD, including CMBs, with comprehensive dental findings. In this article, we detail the protocol for the RAMESSES study (Figure 1).

## Methods

### Study Design and Setting

The RAMESSES study is a multicenter longitudinal prospective cohort study. The purpose of the study is to investigate the relationship between Cnm-positive *S. mutans* and SVD. The study will involve 15 institutes, including the National Cerebral and Cardiovascular Center (NCVC) as the central coordinating center located in Japan. The study will register 230 patients who develop stroke within 1 year after onset and are diagnosed with at least one hemorrhagic lesion involving CMBs or ICH in the deep area detected on T2^*^WI. The primary and secondary outcomes will be assessed at baseline, 6, 12, 18, and 24 months after written informed consent is obtained. We will conduct the assessments according to Table 1 for 2 years. The study protocol has been approved by the Research Ethics Committee of each participating center (NCVC protocol number: M29-133), and this study has been registered with the University Hospital Medical Information Network (UMIN) Clinical Trials Registry (UMIN-CTR: UMIN000045559). Each participant will be provided a detailed explanation of the purpose and potential risks of the study, and written informed consent will be obtained.

**Table 1:** Study schedule.

	**Baseline**	**Follow-up visits**			
		**6 months**	**1 year**	**1.5 years**	**2 years**
Patient characteristics	X				
Physical examinations	X	X	X	X	X
Brain MRI	X		X		X
MoCA	X		X		X
CDR	X				X
GDS	X				
Clinical Frailty Scale	X				X
Laboratory tests	X				
Dental examinations	X				
Assessment of *cnm*	X				
Serum antibody titers against periodontal pathogens	X				

*MRI, magnetic resonance imaging; MoCA, Montreal Cognitive Assessment; CDR, Clinical Dementia Rating; GDS, Geriatric Depression Scale. The mark ‘X' means conduction of assessments*.

### Eligibility Criteria

Inclusion criteria are defined as follows: (1) patients with stroke (cerebral infarction, ICH, or subarachnoid hemorrhage) or transient ischemic attack that developed within the past 365 days; (2) patients over 40 years of age; (3) patients whose modified Rankin Scale (mRS) score is 4 or less; (4) patients who have deep ICH or at least one deep CMB on T2^*^WI performed within the past 365 days; and (5) written informed consent has been obtained from the patients themselves or the proxies. Exclusion criteria are as follows: (1) patients who are contraindicated for MRI examination because of the presence of some metallic implants, such as pacemaker devices; (2) patients with bleeding diathesis; (3) patients who participate in other intervention trials; (4) patients who cannot conduct the Montreal Cognitive Assessment (MoCA) due to severe dementia, deafness, or visual impairment; (5) patients who have no remaining teeth; or (6) patients whose participation is judged as inappropriate by the principal investigator or sub-investigators if a patient is not deemed to have capacity to provide fully informed consent or is too unwell at the time of recruitment (Table 2).

**Table 2:** Eligibility criteria.

**Inclusion criteria**
**Patients must:**
• have been diagnosed with a stroke within the past year
• be ≥40 years of age when providing written informed consent
• have a modified Rankin Scale score in the range of 0–4
• have at least one deep CMB or deep ICH
**Exclusion criteria**
**Patients are ineligible if they:**
• are contraindicated to brain MRI
• have bleeding diathesis
• cannot take MoCA because of severe dementia, auditory disturbance, or visual impairment
• have no teeth

### MRI Evaluation

All 3.0 Tesla MRI examinations will be performed using the following standardized MR imaging protocol. All slices will be taken parallel to the orbitomeatal line from the base of the skull to the vault. The imaging protocol includes T2^*^WI, fluid-attenuated inversion recovery (FLAIR), diffusion-weighted imaging (DWI), and apparent diffusion coefficient. The sequence parameters of T2^*^WI are as follows: voxel size, 0.7 × 0.7 × 4.0 mm; slice gap, 0 mm; flip angle, 20°; field of view, 230 mm; echo time, 12 ms; repetition time, 686 ms. The sequence parameters of FLAIR are as follows: voxel size, 0.7 × 0.7 × 5.0 mm; slice gap, 1 mm; flip angle, 150°; field of view, 230 mm; echo time, 114–117 ms; repetition time, 12,000 ms. The sequence parameters of DWI are not restricted. CMBs and symptomatic ICH will be evaluated using T2^*^WI-MRI. CMBs in deep or lobar areas indicated below will be defined and counted according to the Brain Observer MicroBleed Scale (5) or the Microbleed Anatomical Rating Scale (22). CMBs will be categorized into three groups: (1) deep CMBs defined elsewhere (5, 22), (2) lobar CMBs in the cortical gray or subcortical white matter, and (3) subtentorial CMBs in the cerebellum or brain stem. CMBs in any brain region (any CMB) will also be recorded. Newly developed CMBs will be recorded at follow-up after 1 and 2 years. Symptomatic ischemic stroke (IS) will be evaluated by DWI. Lacunes and WMHs will be evaluated using FLAIR images. A lacune is defined as a supratentorial hypointense lesion 3–15 mm in diameter with a hyperintense rim. Periventricular hyperintensities (PVHs) and deep white matter hyperintensities (DWMHs) will be scored using the Fazekas scale (23).

### Outcomes

The primary outcome is the number of newly developed deep CMBs. The secondary outcomes include (1) new development of lobar, subtentorial, or any type of CMBs; (2) development of symptomatic ICH or IS; (3) change in cognitive function assessed by MoCA and clinical dementia rating (CDR), and in frailty according to the Clinical Frailty Scale version 2.0 (24); (4) major bleeding defined by the International Society on Thrombosis and Hemostasis criteria; (5) all-cause mortality; and (6) serum antibody titers against periodontal pathogens.

### Sample Size Estimates

The sample size was determined by feasibility, but it was ensured to be substantial enough to perform the primary analysis. According to recent studies, the general prevalence rate of Cnm-positive *S. mutans* is 7–20% (19, 25–27). Our previous retrospective study revealed that patients with Cnm-positive *S. mutans* has an incidence rate ratio (IRR) of 13.9 (4.3–44.5) when compared with patients without (20). In this study, the minimum number is 223 subjects (27 subjects with Cnm-positive *S. mutans* and 196 without) based on Cnm-positive *S. mutans* prevalence of 12%, IRR of 4.3 (equivalent to the lower limit of the 95% confidence interval), a significance level of 5%, 90% power, and a follow-up period of 2 years. Therefore, we will recruit 230 subjects with considering a dropout rate of 10%.

### Dental Examination and the Evaluation of Cnm-Positive *S. mutans*

Dental examinations will be conducted by well-trained dentists at each institute. Dental examinations will include (1) checking for the presence of dental caries; (2) dental radiography; (3) probing pocket depth assessed from the free gingival margin to the bottom of the sulcus or pocket; (4) full-mouth bleeding score, which is defined as the number of sites with gingival bleeding on probing divided by the total number of sites per mouth and multiplied by 100 (28); (5) the number of remaining and deficit teeth excluding the third molars; (6) the decayed, missing, and filled teeth (DMFT) index as an indicator of dental history or health (29). Sample collection and detection of Cnm-positive *S. mutans* will be performed using the following standardized protocol: oral saliva and dental plaque specimens will be collected from the patients using Seed-swab Type ⋎3 (Eiken Chemical, Tokyo, Japan). These samples will be inoculated on Mitis-Salivarius medium with bacitracin (MSB, 100 U/ml; Sigma-Aldrich, St. Louis, MO, USA) and 15% sucrose (MSB agar) and anaerobically incubated at 37°C for 48 h. The *S. mutans* colonies will be isolated and cultured in Brain Heart Infusion Broth (Difco Laboratories, Detroit, MI, USA) at 37°C for 24 h. Then, DNA from each strain will be extracted and analyzed by polymerase chain reaction using species-specific MKD primers to identify *S. mutans* isolates (27) and *cnm* primers to detect the *cnm* gene (16). A collagen-binding assay with type I collagen will be conducted to examine the collagen-binding activity of each isolated *S. mutans* strain, according to the method described elsewhere (30), with some modifications (27). The activity tests will be performed under fixed conditions using 1 mg of type I collagen and 1 × 10^10^ bacterial cells. The activity for each strain is expressed as a percentage compared with the positive control *S. mutans* TW871, which has 100% binding activity to type I collagen (27). These experiments will be conducted by a researcher who is blinded to the clinical information.

### Assessment of Serum Antibody Titers Against Periodontal Pathogens

Serum samples will be collected during baseline evaluation to measure serum antibody titers against nine bacteria responsible for periodontal diseases by enzyme-linked immunosorbent assay, as previously reported (31). The nine bacteria are *Porphyromonas gingivalis* (*P. gingivalis*), *Aggregatibacter actinomycetemcomitans, Eikenella corrodens, Fusobacterium nucleatum, Prevotella nigrescens, Prevotella intermedia, Treponema denticola, Tannerella forsythia*, and *Campylobacter rectus*.

### Psychological Examinations

The cognitive function and mental status of the subjects will be evaluated in each institute by well-trained neurologists or psychologists who are familiar with CDR (at baseline and 2 years), MoCA (at baseline, 1 and 2 years), and geriatric depression scale (at baseline).

### Clinical Frailty Scale

The frailty at baseline and 2 years (32) will be assessed in each institute by well-trained neurologists using the Clinical Frailty Scale version 2.0 (24).

### Data Management

An electronic data capture system, Research Electronic Data Capture (REDCap; Vanderbilt University, Nashville, TN, USA) (33), will be used for the collection and management of all data. Brain MRI data will be sent to the Central Evaluation Committee, and the data will be assessed by three neurologists belonging to the committee. Dental findings will be evaluated and stored by dentists in the Department of Pediatric Dentistry at Osaka University. All data will be collected and integrated at the NCVC. The data managers will perform quality control at each step of data handling to ensure the reliability of all data related to the study.

### Statistical Analyses

For the primary outcome, the incidence of newly developed CMBs will be compared between patients with and without Cnm-positive *S. mutans* using a quasi-Poisson regression model, adjusted for age, sex, risk factors, antithrombotic medication, history of heart disease, history of cerebrovascular diseases, and dental findings. For the secondary outcomes, which include the incidence of symptomatic ICH or IS, all-cause mortality, and total hemorrhagic events, the survival time curves will be derived for each group using the Kaplan-Meier method and the survival rates will be compared between the two groups using the log-rank test and Cox proportional hazard models. The changes of scores based on frailty scale scoring, the cognitive tests from baseline to 2 years, and the antibody titers against periodontal pathogens at baseline will be compared between patients with and without Cnm-positive *S. mutans* using generalized linear mixed models for repeated measurements based on data distributions. Statistical analysis will be conducted using SAS version 9.4 (SAS Institute, Cary, USA).

### Study Organization and Funding

The study will be conducted at 15 Japanese institutes, including the NCVC, and is supported by funding from Grant-in-Aid for Early-Career Scientists, KAKEN (YH: 21K16961), and the BMS/Pfizer Japan Thrombosis Investigator Initiated Research Program (MI: CV185656).

## Discussion

The objective of the RAMESSES study, a multicenter longitudinal prospective cohort study, is to assess whether Cnm-positive *S. mutans* affects the development of deep CMBs, as the primary outcome. The secondary outcomes include assessments of the effects of Cnm-positive *S. mutans* on the new development of lobar, subtentorial, or any type of CMBs, symptomatic ICH or IS, the changes in cognitive function or frailty, major bleeding, all-cause mortality, and antibody titers against periodontal pathogens.

**Figure 2:**
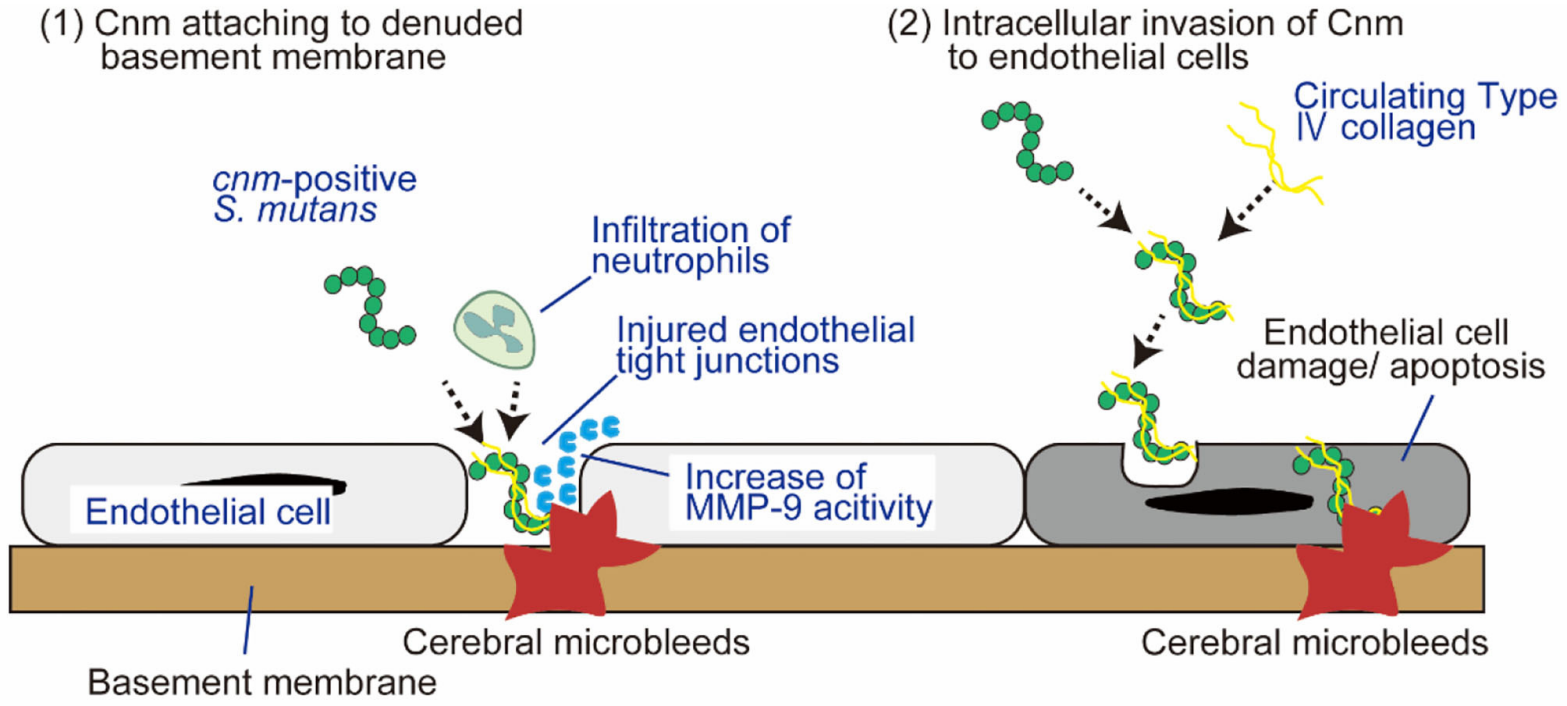
Two plausible mechanisms by which Cnm is involved in blood-brain barrier injury and subsequent development of CMBs. There are two plausible mechanisms by which Cnm is involved in BBB injury and subsequent development of CMBs: (1) Cnm-positive *S. mutans* attaches to denuded basement membrane facilitated by endothelial tight junction dehiscence. (2) Cnm-positive *S. mutans* directly invades endothelial cells. See text for details. BBB, blood-brain barrier; MMP-9, matrix metalloproteinase-9.

Cnm-positive *S. mutans* causes SVD, including CMBs and ICH, due to the collagen-binding activity, which mediates binding to type I collagen in teeth (27) and to type IV collagen (34) and laminin (35) in the basement membrane of cerebral blood vessels. Cnm-positive *S. mutans* binds strongly to the dentin tooth layer composed of type I collagen, which mediates the entry of *S. mutans* into the bloodstream by promoting the development of carious lesions in the periodontal space (34). After the induction of bacteremia, there are two plausible mechanisms by which Cnm is involved in BBB injury and subsequent development of CMBs. First, Cnm-positive *S. mutans* can adhere and attach to the denuded basement membrane via endothelial tight junction dehiscence, and the migration of circulating neutrophils to the lesion subsequently activates local inflammation with the secretion of matrix metalloproteinase (MMP)-9 that eventually exacerbates the BBB permeability (17). Second, Cnm-positive *S. mutans* has the ability to invade endothelial cells, which specifically requires Cnm for adherence and intracellular invasion (36–38). Once Cnm binding to circulating type IV collagen mediates attachment to the host endothelial surface, host cell signaling cascades are triggered that lead to the internalization of Cnm-positive *S. mutans*, the activation of pro-inflammatory responses, and eventually apoptosis of endothelial cells (36, 39) (Figure 2). BBB dysfunction not only induces red blood cell extravasation, forming CMBs and ICH, but also may aggravate the homeostasis of the internal environment in the brain with reduced delivery of glucose and other nutrients, along with impaired clearance of waste products and metabolites through efflux transporters, all of which can possibly lead to cognitive impairment (40).

*P. gingivalis* is the main pathogen for periodontitis (41). Adherence of *P. gingivalis* to endothelial cells activates coagulation factors and protease-activated receptors 1 and 4 expressed on the surface of platelets (41). In addition, periodontal disease-induced disruption of the gingival epithelial barrier mediates bacteremia onset by facilitating the entry of oral bacteria, including *S. mutans*, into systemic circulation (42). Thus, oral carriage of *P. gingivalis* is possibly implicated in SVD pathology due to thrombotic occlusion and BBB disruption induced by endothelial damage and inflammation (43).

In conclusion, Cnm-positive *S. mutans* probably plays a critical role in the development of SVD, including CMBs and ICH, based on previous findings reported in basic and clinical retrospective observational studies. The RAMESSES study is expected to establish a firm understanding of the pathophysiology of Cnm-positive *S. mutans* for SVD as the “brain-oral axis”, which will guide the future development of novel prophylactic strategies against Cnm-positive *S. mutans*-induced SVD and vascular cognitive impairment.

## Ethics Statement

The protocol and related documents have been approved by the Research Ethics Committee at the National Cerebral and Cardiovascular Center (approval number: M29-133). The patients/participants provided their written informed consent to participate in this study.

## Author Contributions

SH and YH wrote the manuscript and contributed to the study protocol. SS contributed to the study design and revised the manuscript. MT leads the statistical analysis. ST, YY, SI, and YMi discussed the study protocol. NO and YMo are responsible for the standardization of the MRI protocol. RN and KN contributed to dental examinations and expertise. MI conceived the study design, wrote the manuscript, and worked on revisions. All authors contributed to the article and approved the submitted version.

## Funding

This study receives funding from Bristol-Myers Squibb (CV185656). The funder is not involved in the study design, collection, analysis, interpretation of data, the writing of this article or the decision to submit it for publication.

## Conflict of Interest

MI reports personal fees from Daiichi Sankyo, Eisai, and Bayer, and grants from Panasonic, Bristol-Myers Squibb, and Otsuka Pharmaceutical, outside the submitted work. The remaining authors declare that the research was conducted in the absence of any commercial or financial relationships that could be construed as a potential conflict of interest.

## Publisher's Note

All claims expressed in this article are solely those of the authors and do not necessarily represent those of their affiliated organizations, or those of the publisher, the editors and the reviewers. Any product that may be evaluated in this article, or claim that may be made by its manufacturer, is not guaranteed or endorsed by the publisher.
